# Sertoli Cells Improve Myogenic Differentiation, Reduce Fibrogenic Markers, and Induce Utrophin Expression in Human DMD Myoblasts

**DOI:** 10.3390/biom11101504

**Published:** 2021-10-12

**Authors:** Laura Salvadori, Sara Chiappalupi, Iva Arato, Francesca Mancuso, Mario Calvitti, Maria Cristina Marchetti, Francesca Riuzzi, Riccardo Calafiore, Giovanni Luca, Guglielmo Sorci

**Affiliations:** 1 Department of Translational Medicine, University of Piemonte Orientale, 28100 Novara, Italy; laura.salvadori@uniupo.it; 2 Interuniversity Institute of Myology (IIM), 06132 Perugia, Italy; sarac.chiappalupi@gmail.com (S.C.); francesca.riuzzi@unipg.it (F.R.); 3 Department of Medicine and Surgery, University of Perugia, 06132 Perugia, Italy; iva.arato@libero.it (I.A.); francesca.mancuso@unipg.it (F.M.); mario.calvitti@unipg.it (M.C.); maria.marchetti@unipg.it (M.C.M.); riccardo.calafiore@unipg.it (R.C.); 4 Consorzio Interuniversitario Biotecnologie (CIB), 34127 Trieste, Italy; 5 Centro Biotecnologico Internazionale di Ricerca Traslazionale ad Indirizzo Endocrino, Metabolico ed Embrio-Riproduttivo (CIRTEMER), 06132 Perugia, Italy; 6 Centro Universitario di Ricerca sulla Genomica Funzionale (CURGeF), 06132 Perugia, Italy

**Keywords:** duchenne muscular dystrophy (DMD), Sertoli cell, utrophin, heregulin β1, myoblast differentiation, fibrosis, myofibroblast transdifferentiation

## Abstract

Duchenne muscular dystrophy (DMD) is an X-linked disease caused by mutations in *DMD* gene translating in lack of functional dystrophin and resulting in susceptibility of myofibers to rupture during contraction. Inflammation and fibrosis are critical hallmarks of DMD muscles, which undergo progressive degeneration leading to loss of independent ambulation in childhood and death by early adulthood. We reported that intraperitoneal injection of microencapsulated Sertoli cells (SeC) in dystrophic mice translates into recovery of muscle morphology and performance thanks to anti-inflammatory effects and induction of the dystrophin paralogue, utrophin at the muscle level, opening new avenues in the treatment of DMD. The aim of this study is to obtain information about the direct effects of SeC on myoblasts/myotubes, as a necessary step in view of a translational application of SeC-based approaches to DMD. We show that (i) SeC-derived factors stimulate cell proliferation in the early phase of differentiation in C2C12, and human healthy and DMD myoblasts; (ii) SeC delay the expression of differentiation markers in the early phase nevertheless stimulating terminal differentiation in DMD myoblasts; (iii) SeC restrain the fibrogenic potential of fibroblasts, and inhibit myoblast-myofibroblast transdifferentiation; and, (iv) SeC provide functional replacement of dystrophin in preformed DMD myotubes regardless of the mutation by inducing heregulin β1/ErbB2/ERK1/2-dependent utrophin expression. Altogether, these results show that SeC are endowed with promyogenic and antifibrotic effects on dystrophic myoblasts, further supporting their potential use in the treatment of DMD patients. Our data also suggest that SeC-based approaches might be useful in improving the early phase of muscle regeneration, during which myoblasts have to adequately proliferate to replace the damaged muscle mass.

## 1. Introduction

Duchenne muscular dystrophy (DMD) is a recessive X-linked lethal disease affecting one over 3600–5000 live male births [1,2]. DMD is due to mutations in the dystrophin gene (*DMD*) leading to lack of a functional protein and subsequent inability to recruit the dystrophin-associated protein complex (DAPC) at the sarcolemma resulting in susceptibility of myofibers to rupture during contraction [3]. Muscles of DMD patients or experimental models of DMD show progressive necrosis of the myofibers, chronic inflammation, and reactive regeneration, which lead to exhaustion of muscle precursor cell pool and replacement of myofibers with fibrous and fatty tissues [3,4]. Inflammation and fibrosis are the most critical hallmarks of DMD muscles, responsible of the onset and the progression of the pathology [5,6]. DMD patients experience loss of independent ambulation by their teenage years, and die in the early adulthood due to cardiac and respiratory failure [3,7]. A wide spectrum of therapeutic approaches to DMD have been explored in the last decades and many of them are currently under clinical evaluation, although several limitations, such as unsatisfying muscle recovery, the need for immunosuppression, and relevant adverse effects encourage for further investigation [8,9,10]. The standard therapy to DMD still remains the use of glucocorticoids despite their limited efficacy and undesired side effects [10].

We set up a therapeutic approach [11,12] based on the peculiar properties of Sertoli cells (SeC), a cell type of the seminiferous tubules of the testis in which they favor the maturation of developing germ cells and protect them against the host immune system [13]. Besides creating a physical barrier (the blood–testis barrier), SeC secrete a plethora of trophic and immunomodulatory factors that confer to these cells the ability to survive long time after engraftment, and protect allo- and xenogenic engraftments of tissues and organs [14,15,16]. SeC isolated from specific pathogens free (SPF) pre-pubertal pigs were encapsulated in highly biocompatible alginate-based microcapsules (MC-SeC) and injected into the peritoneal cavity of *mdx* mice, an experimental model of DMD, in the absence of pharmacological immunosuppression [11,12]. A single injection of MC-SeC resulted in amelioration of muscle morphology and performance as a consequence of the secretion by SeC, from the peritoneal cavity into the bloodstream, of anti-inflammatory factors and heregulin β1, an inducer of the dystrophin paralogue, utrophin. Short- and long-term beneficial effects of MC-SeC treatment (i.e., reduction of inflammatory infiltrate, re-expression of utrophin at the sarcolemma, and reduced necrosis of myofibers) have been reported in several muscles, including the diaphragm, thus opening new perspectives in the treatment of DMD [11,12]. Here, we have investigated the effects of SeC on muscle precursor cells, with particular regards to human healthy and DMD myoblasts/myotubes as a necessary step in view of a potential application of SeC-based treatments to DMD.

## 2. Materials and Methods

A detailed Key resources table (Table A1) is reported in Appendix A.

### 2.1. Sertoli Cell Isolation, Purification, and Characterization

SeC were isolated from testes of Large White neonatal pigs, according to established methods modified in our laboratory [12,17]. Briefly, after removal of the fibrous capsule the testes were finely chopped and digested enzymatically with a solution of trypsin and DNase I followed by digestion with collagenase P. The pellet was passed through a 500-mm stainless steel mesh and resuspended in glycine to eliminate residual Leydig and peritubular cells. The resulting SeC were cultured in HAM’S F12 supplemented with 0.166 nM retinoic acid and 1% Insulin-Transferrin-Selenium (ITS) + Premix in 95% air/5% CO_2_ at 37 °C. Detection of anti-Müllerian hormone (AMH, unique prepubertal SeC marker), insulin-like3 (INSL3, Leydig cells marker), alpha-smooth muscle actin (ASMA, peritubular cells marker), and protein gene product 9.5 (PGP9.5, gonocyte and spermatogonial cells marker) were performed [12,17].

### 2.2. Sertoli Cell-Conditioned Medium

SeC were seeded in complete medium composed of HAM’S F12 supplemented with 0.166 nM retinoic acid and 1% of Insulin-Transferrin-Selenium (ITS) + Premix. After 3 days of culture, complete medium was replaced with high-glucose Dulbecco’s modified Eagle’s medium (HG-DMEM) supplemented with 100 U/mL penicillin and 100 mg/mL streptomycin (P/S) without any other nutritional sources (1 mL/1.0 × 10^6^ SeC) for 72 h. Thereafter, SeC-conditioned medium was collected and centrifuged at 2500× *g* 30 min to discard cell debris. The supernatant was recovered and added with 2% horse serum (HS) to obtain SeC-conditioned differentiation medium (SeC-DM). Unconditioned differentiation medium (U-DM) was obtained using the same procedure in the absence of SeC. Freshly-prepared SeC-DM or U-DM were used in experiments with C2C12 myoblasts.

### 2.3. Cell Culture

Murine C2C12 myoblasts (CRL-1772) and human WI-38 fibroblasts (CCL-75), obtained from the American Type Culture Collection (Manassas, VA, USA), were cultured in growth medium composed of HG-DMEM supplemented with 20% fetal bovine serum (FBS) and P/S. C2C12 myoblasts were seeded 2.9 × 10^4^ cells/cm^2^, cultured for 24 h in GM, and then shifted to SeC-DM or U-DM for the indicated times. Where indicated, myoblasts were cultured in SeC-DM or U-DM in the presence of TGF (transforming growth factor)-β (5 ng/mL) for 24 h in order to induce myoblast-myofibroblast transdifferentiation. Human healthy and DMD primary myoblasts were obtained from Telethon Network of Genetic Biobanks and the Myobank-AFM of the Institut de Myologie. Human myoblasts were cultured in Skeletal Muscle Cell Growth Medium (Promo Cell) supplemented with 15% FBS and P/S. To obtain myotubes, myoblasts were seeded 2.9 × 10^4^ cells/cm^2^ in growth medium and, after 24 h, switched to HG-DMEM supplemented with 5% HS, insulin (10 µg/mL), apotransferrin (100 µg/mL), gentamicin sulfate (50 µg/mL), and L-Glutamine (1%) (human differentiation medium, huDM). WI-38 fibroblasts or human myoblasts or myotubes were co-cultured in the absence or presence of freshly isolated SeC (2.0 × 10^5^ SeC/mL) as indicated by the use of transwells (pores 0.4 µm, Falcon). In some experiments, human DMD myotubes were co-cultured with or without SeC in the absence or presence of anti-heregulin 1β blocking antibody (2 μg/mL) or the same amounts of non-immune IgG added to the upper chamber of the transwells for 48 h. Human DMD myotubes pretreated with the ErbB receptor tyrosine kinase inhibitor, PD158780 (100 nM), or vehicle (DMSO) for 20 min were cultured in SeC-DM or U-DM in the absence or presence of PD158780 for additional 24 h. Cells were maintained in a humidified atmosphere containing 5% CO_2_ at 37 °C.

### 2.4. May–Grünwald/Giemsa Staining and Morphometric Evaluations

Fusion indexes, total nuclei and nuclei/myotube ratios were determined after May–Grünwald/Giemsa staining, as reported [17,18]. Briefly, C2C12 and human primary myoblasts were fixed in cold absolute methanol for 8 min and stained with May–Grünwald. After 3 min, Sorensen’s phosphate buffer (0.067 M, pH 6.8) was added for 4 min. Cells were then stained with Giemsa solution diluted in Sorensen’s phosphate buffer 1:10 for 12 min. Cells were dried and observed with an Olympus BX51 microscope (Olympus, Milan, Italy). Evaluations were performed on cells acquired at 10× magnification in five randomly selected fields/well using Image J software (ImageJ v1.8.0_172, National Institutes of Health, USA). The fusion indexes were calculated as the percentages of nuclei inside myotubes (i.e., syncytia containing at least three nuclei) versus the total number of nuclei.

Myotube diameters were measured after immunofluorescence for myosin heavy chain (MyHC)-II (see paragraph 2.5), as described [18]. Briefly, 10 randomly chosen fields of MyHC-II stained images were acquired at 20× magnification for each condition and three different measurements were performed along the longitudinal axis of each myotube using Image J software. Results were expressed as percentages of average myotube diameters with respect to control myotubes.

### 2.5. Immunofluorescence

Human myoblasts were seeded on glass coverslips pretreated with gelatin, and co-cultured with or without SeC for 48 h. Myoblasts were fixed in cold absolute methanol for 8 min and permeabilized for 10 min with 0.4% Triton X-100 in phosphate-buffered saline (PBS). Then, samples were treated with blocking buffer (BB; 3% bovine serum albumin (BSA) and 1% glycine in PBS) for 1 h, and incubated overnight (O.N.) at 4 °C with an anti-utrophin (1:10) or anti-α-dystroglycan (1:20) primary antibody in 3% BSA in PBS. The day after, samples were washed with 0.01% Tween-20 in PBS (T-PBS) and incubated with an anti-mouse Alexa Fluor 594-conjugated antibody diluted 1:100 in 3% BSA in PBS for 1 h. Nuclei were counterstained with 4′,6-diamidino-2-phenylindole (DAPI). Immunofluorescence for MyHC-II was performed to evaluate myotube diameters, as described [19]. For 5-bromo-2′-deoxyuridine (BrdU) staining, C2C12 myoblasts were seeded on pretreated glass coverslips 1.3 × 10^4^ cells/cm^2^ and the day after switched to U-DM or SeC-DM. After 24 h, BrdU (10 µM) was added for 1 h, and the cells were fixed in cold absolute methanol for 10 min and permeabilized for 5 min with 0.1% Triton X-100 in PBS. Samples were incubated with hydrochloric acid (HCl, 2N) for 30 min and washed with borate buffer (0.1 M, pH 8.3) followed by PBS, before incubation with an anti-BrdU primary antibody (1:50) in 3% BSA in PBS for 1 h at room temperature (RT). After washes in T-PBS and PBS, samples were incubated with the secondary anti-mouse Alexa Fluor 488-conjugated antibody (1:100 in T-PBS) for 1 h at RT. Nuclei were counterstained with DAPI. Samples were mounted with fluorescent mounting medium and viewed by an epifluorescence microscope (Leica DMRB, Milan, Italy) equipped with a digital camera.

### 2.6. Western Blotting

Cells were lysed in protein extraction buffer, and equal amounts of total protein extract (30 μg) were resolved by SDS-PAGE for Western blotting analysis, as described in [19]. Primary and secondary antibodies used are reported in Appendix B (Table A2).

### 2.7. Real-Time PCR

Total RNA from human primary myoblasts was extracted using TRIsure ™ reagent (Bioline) following the manufacturer’s instructions. Reverse-transcription was performed using PrimeScript ™ RT reagent kit (Takara Bio Europe, Saint-Germain-en-Laye, France). Real-time PCR analysis was performed on Stratagene Mx3000P using 5 × HOT FIREPol EvaGreen qPCR Mix Plus (ROX) ready-to-use solution (Solis BioDyne, Tartu, Estonia) using the primers reported in Appendix C (Table A3). Calculation was performed with a dedicated software in comparison with a standard gene (GAPDH).

### 2.8. Cytofluorimetric Analysis

Cells were cultured in 6-well plates. After treatment, cells that were floating in the culture medium were harvested, washed with PBS, and added with ipotonic propidium iodide solution (PI; 0.1% sodium citrate, 0.1% Triton X-100, and 50 mg/l propidium iodide). These cells suspended in PI were pooled with the adherent cells in the respective well previously washed with PBS. Reunited cells were incubated with PI for 30 min at 4 °C. Cell cycle analysis was performed by flow cytometry using fluorescence-activated cell sorting (FACS, Coulter Epics XL-MLC with a beam of light from a 488 nm argon laser).

The percentages of apoptotic cells were evaluated by an annexin V/propidium iodide-based commercial kit (Annexin V-FITC apoptosis detection kit; BioVision Inc., Milpitas, CA, USA) following the manufacturer instructions. Briefly, adherent cells were gently trypsinized, washed, and incubated with annexin V-FITC and propidium iodide for 5 min at RT before being quantified by flow cytometry using FL1 and FL2 signal detectors.

### 2.9. DCFH-DA Assay

The DCFH-DA (dichlorodihydrofluorescein diacetate) method was used to detect intracellular ROS. C2C12 myoblasts were cultured in U-DM or SeC-DM for 24 h, and DCFH-DA (30 μM) was added to the medium for 30 min at 37 °C. The fluorescence of 2′,7′-dichlorofluorescein (DCF) was detected (485 nm excitation, 535 nm emission) using a Titertek Fluoroscan II (Flow Laboratories, McLean, VA, USA).

### 2.10. Statistical Analysis

Quantitative data are presented as means ± SD (standard deviation) or SEM (standard error of the mean) of at least three independent experiments. Counts were performed by three independent operators blind to the treatments. Representative experiments and images are shown unless stated otherwise. Statistical analysis was performed using two-tailed, unpaired *t* test. P values lower than 0.05 were considered statistically significant. Statistical data were processed by IBM^®^ SPSS^®^ Statistics Version 18 software (IBM, Armonk, NY, USA).

## 3. Results

### 3.1. SeC Stimulate Myoblast Proliferation without Affecting the Myogenic Potential

**Figure 1:**
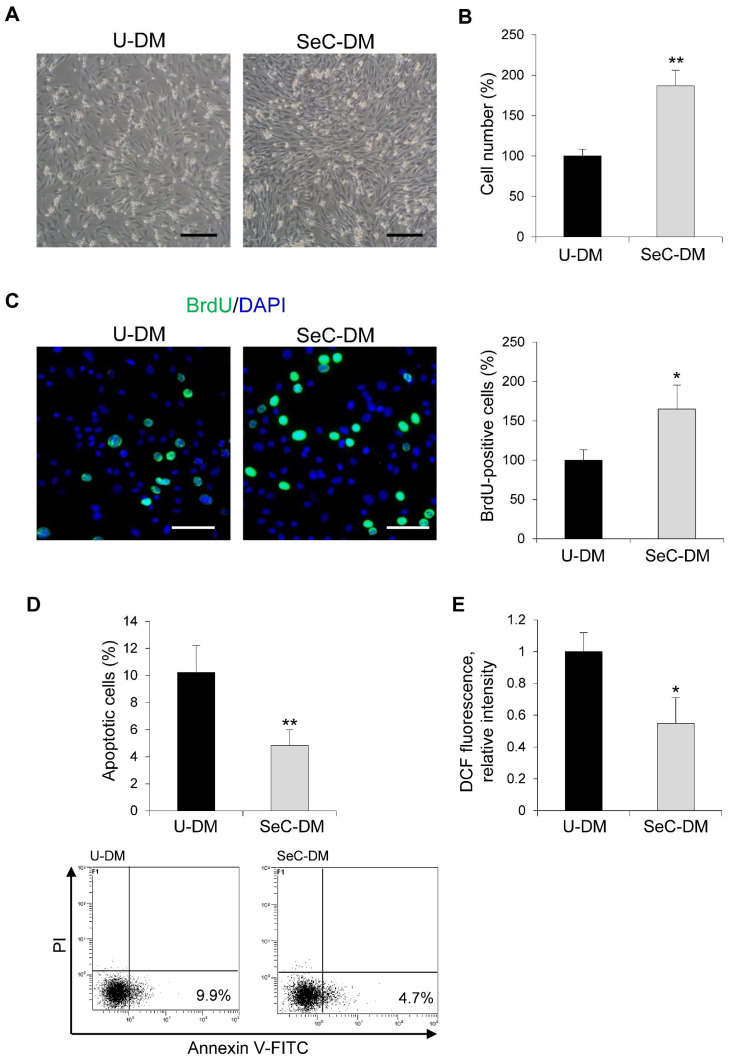
SeC increase C2C12 myoblast numbers in the early phase of the differentiation process. (**A**–**E**) C2C12 myoblasts were cultivated for 24 h in the presence of unconditioned differentiation medium (U-DM) or DM conditioned by Sertoli cells (SeC-DM). (**A**) Myoblast cultures were observed by phase contrast microscopy (representative images). (**B**) Cell count and (**C**) BrdU assay were performed to evaluate the percentage differences in the number of cells or proliferating cells, respectively. Reported are representative merged images of BrdU (green) and DAPI (blue) used to counterstain nuclei. (**D**) The percentages of apoptotic cells were determined by FACS analysis after annexin V-FITC and propidium iodide (PI) staining. Representative scatterplots are reported. (**E**) The 2′,7′-dichlorofluorescein (DCF) fluorescence was measured to evaluate the intracellular levels of reactive oxygen species (ROS). Results are means of three independent experiments (±SD). * and **, significantly different from U-DM (*p* < 0.05 and *p* < 0.01, respectively). Scale bars, 200 µm (**A**) and 100 µm (**C**).

The direct effects of SeC on myoblasts have never been reported so far. C2C12 myoblasts cultured in growth medium (GM) were switched to unconditioned (U-DM) or SeC-conditioned (SeC-DM) differentiation medium for different times. After 24 h, myoblasts cultured in SeC-DM showed increased cell numbers in comparison with control myoblasts, as observed by inverted phase contrast microscopy (Figure 1A) and evaluated by cell count (Figure 1B), suggesting that SeC-released factors increase cell proliferation and/or reduce cell death. Indeed, the BrdU incorporation assay showed a ~65% increase in the percentage of BrdU-positive (i.e., replicating) cells (Figure 1C), and cytofluorimetric analysis after annexin V/propidium iodide staining showed a concomitant ~53% reduction in the apoptotic extent (Figure 1D), in the presence of SeC-DM compared with U-DM. Moreover, differentiating myoblasts cultured with SeC-DM were characterized by reduced oxidative stress since we found 45.0 ± 14% reduction in DCF fluorescence relative intensity, which is a measure of intracellular production of reactive oxygen species (ROS), compared with control myoblasts (Figure 1E).

**Figure 2:**
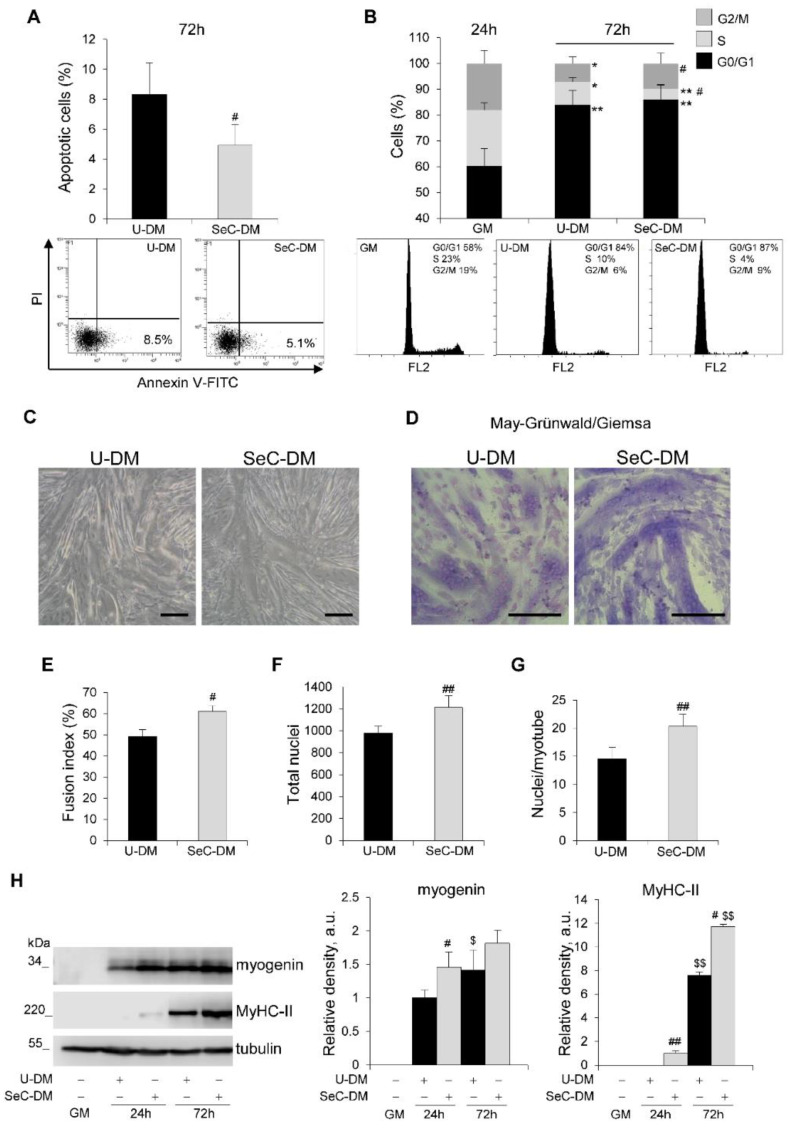
SeC promote myogenic terminal differentiation. (**A**–**G**) C2C12 myoblasts were cultivated either in growth medium (GM) for 24 h or in unconditioned medium (U-DM) or DM conditioned by Sertoli cells (SeC-DM) for 72 h. FACS analysis was performed to evaluate the percentages of apoptotic cells after annexin V-FITC and propidium iodide (PI) staining at 72 h in DM. Representative scatterplots are reported (**A**). The cell cycle in GM (24 h) and DM (72 h) was evaluated by FACS analysis. Representative (**B**). Reported are representative images of phase contrast microscopy (**C**) and May–Grünwald/Giemsa staining (**D**) of myoblasts cultured in U-DM or SeC-DM for 72 h. The fusion indexes (**E**), the total nuclei/field (**F**), and the average numbers of nuclei/myotube (**G**) in myoblasts cultured as in (**C**,**D**) were determined. (**H**) C2C12 myoblasts were cultivated either in GM for 24 h or in U-DM or SeC-DM for 24 h or 72 h, as indicated. Cells were lysed and the expression of myogenin and myosin heavy chain (MyHC)-II were analyzed by WB. Representative images are shown. The average relative densities of the bands with respect to tubulin bands, used as house-keeping protein, are reported. Results are means ± SD (**A**,**B**) or ±SEM (**E**–**H**) of three independent experiments. * and **, significantly different from GM (*p* < 0.05 and *p* < 0.01, respectively); # and ##, significantly different from U-DM at the same time-point (*p* < 0.05 and *p* < 0.01, respectively); $ and $$, significantly different from the same treatment at 24 h (*p* < 0.05 and *p* < 0.01, respectively). Scale bars (**C**,**D**), 200 µm.

Apoptotic events increase physiologically during the myogenic differentiation process, serving the critical function of removing excess myoblasts and limiting muscle mass [20]. Thus, we evaluated myoblast cultures at 72 h in DM by cytofluorimetric analysis and we found again a reduced (~41% reduction) apoptosis extent (Figure 2A) together with a strong reduction (~65%) of cells in the S phase in the presence of SeC-DM compared to U-DM (Figure 2B), compatible with a more differentiating cell population. At the same time-point, myoblasts cultured with SeC-DM showed an increased extent of fusion into myotubes, as observed by phase contrast microscopy (Figure 2C) and confirmed by the evaluation of the fusion indexes (i.e., the percentages of nuclei residing in syncytia containing ≥3 nuclei) after May–Grünwald/Giemsa staining (Figure 2D,E). Moreover, at 72 h the total nuclei/field ratio was increased in the presence of SeC-DM (Figure 2F), in accordance with the stimulated proliferation and the reduced apoptosis extent observed at 24 h in this condition (Figure 1), and higher numbers of nuclei/myotube were found in SeC-DM compared with U-DM (Figure 2G). Western blot analysis revealed increased amounts of the muscle-specific transcription factor and terminal differentiation marker, myogenin, at 24 h, and increased amounts of the structural protein, myosin heavy chain (MyHC)-II, at 24 h (when MyHC-II was undetectable in U-DM-cultured myoblasts) and 72 h, in the presence of SeC-DM (Figure 2H).

Altogether, these results indicated that factors secreted by SeC stimulate the proliferation of muscle precursor cells and protect them against differentiation-associated apoptosis thus increasing their numbers (Figure 1), and improve their myogenic potential once exposed to differentiating conditions (Figure 2). This was further confirmed by an experiment in which C2C12 myoblasts were co-cultured with high doses of SeC (microencapsulated SeC, MC-SeC) for 72 h, resulting in increased cell numbers and almost absence of myotube formation, and then cultured for additional 72 h after removal of MC-SeC, resulting in a dramatic fusion into myotubes that reached the extent of myoblasts co-cultured with empty microcapsules [17].

### 3.2. SeC Favor Myotube Elongation and Myosin Heavy Chain Expression in Differentiating DMD Myoblasts

**Figure 3:**
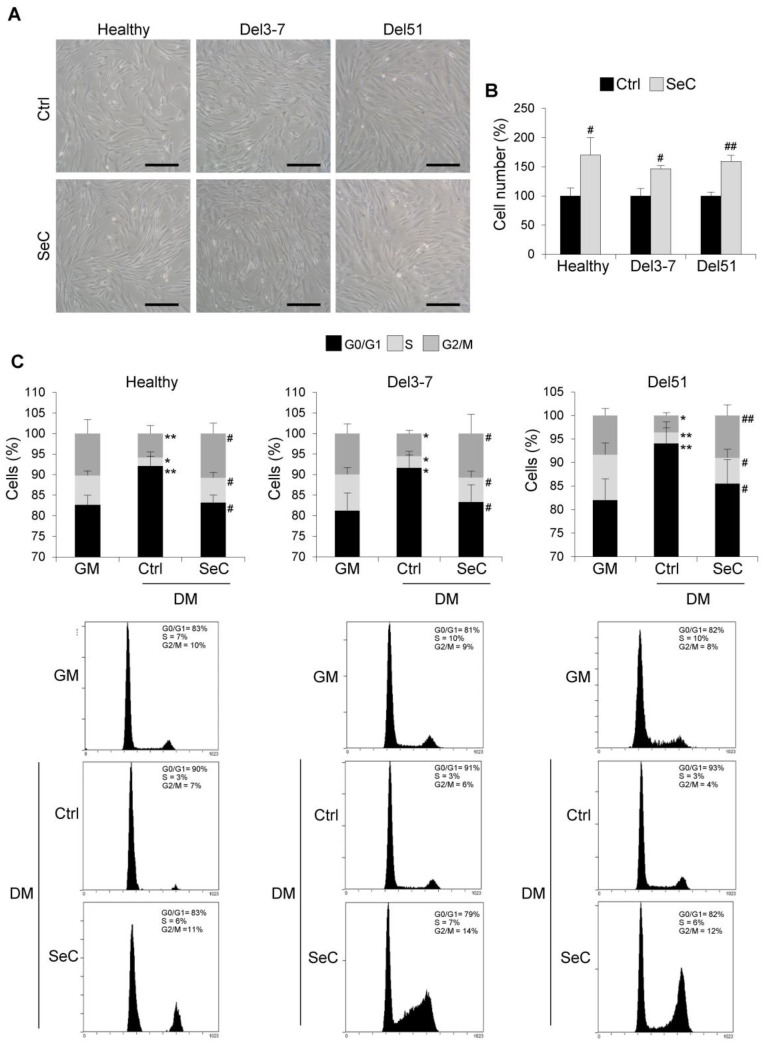
SeC promote cell proliferation in the early phase of the differentiation process in human healthy and DMD myoblasts. (**A**–**C**) Human myoblasts derived from healthy donor and DMD (Del3-7 and Del51) patients with different mutations were co-cultured in DM with or without freshly-isolated SeC (2.0 × 10^5^ SeC/mL) using 0.4 μm BD transwells, for 24 h. (**A**) Reported are representative phase contrast microscopy images. (**B**) Cell counts were performed to determine the percentage differences in cell numbers. (**C**) FACS analysis was performed to evaluate the percentages of cells in the different phases of the cell cycle in DM at 24 h in comparison with untreated cells cultured for 24 h in GM. Results are means of three independent experiments (±SD). * and **, significantly different from GM (*p* < 0.05 and *p* < 0.01, respectively). # and ##, significantly different from Ctrl (*p* < 0.05 and *p* < 0.01, respectively). Scale bars (**A**), 200 µm.

Therefore, we investigated the effects of SeC on human healthy and DMD myoblasts with different mutations. Human myoblasts derived from muscle biopsies were co-cultured with freshly purified SeC (2.0 × 10^5^ SeC/mL) with the use of 0.4 µm pore size transwells. Deletion of exons 3–7 (Del3-7) and deletion of exon 51 (Del51) were investigated. At 24 h in DM, in healthy and DMD myoblasts higher numbers of cells could be seen by inverted phase contrast microscopy in the presence of SeC compared with control cultures (Figure 3A), which was confirmed by cell counts (increase in cell numbers ranging from 46.7 to 70.0%) (Figure 3B). Cytofluorimetric analysis at this time-point showed no significant differences in the apoptosis extents, which were found very low (about 3–5%) in both healthy and DMD myoblasts irrespective of the presence of SeC (not shown). The analysis of the cell cycle at 24 h in DM showed reduced percentages of cells in S and G2/M phases, and increased percentages in G0/G1 phase compared with GM conditions in control healthy and DMD myoblasts, typical of myoblasts entering the differentiation program (Figure 3C). However, co-culture with SeC for 24 h in DM translated into increased percentages of cells in S and G2/M phases, and reduced percentages in G0/G1 phase compared with untreated myoblasts at the same time-point (Figure 3C).

**Figure 4:**
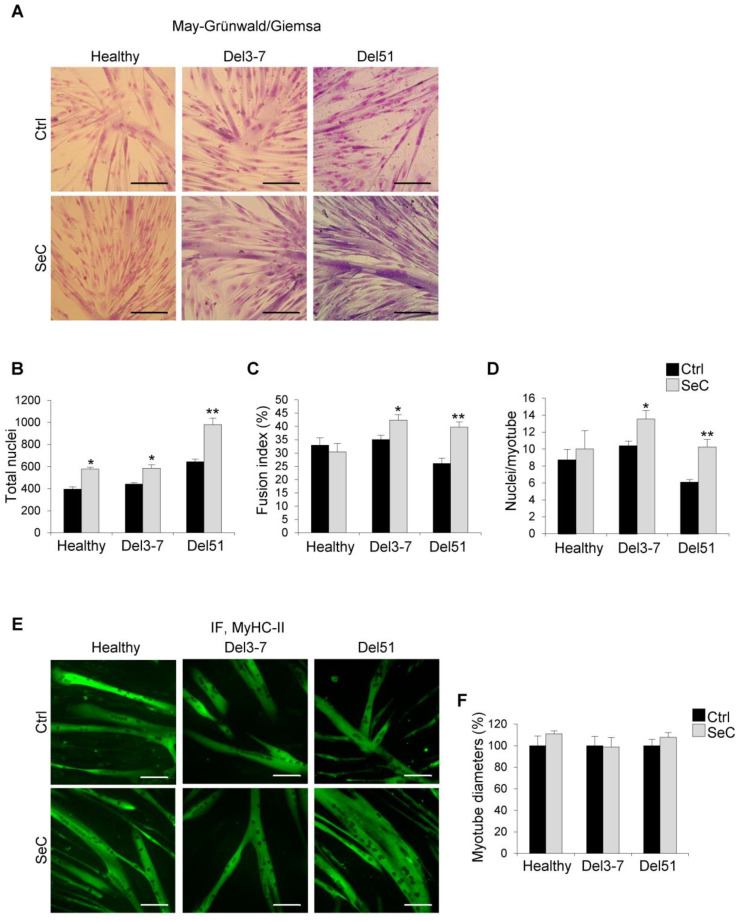
Human DMD myoblasts treated with SeC show improved myogenic differentiation. (**A**–**F**) Human myoblasts derived from healthy donor and DMD (Del3-7 and Del51) patients were co-cultured in DM with (SeC) or without (Ctrl) freshly-isolated SeC (2.0 × 10^5^ SeC/mL) using 0.4 μm BD transwells, for 6 days. After May–Grünwald/Giemsa staining (**A**; representative images), the numbers of total nuclei/field (**B**), the fusion index (**C**), and the numbers of nuclei/myotube (**D**) were determined. After immunofluorescence staining for MyHC-II (green) (**E**; representative images), the percentage changes in myotube diameters were determined (**F**). Results are means of three independent experiments (±SEM). * and **, significantly different from internal control (Ctrl) (*p* < 0.05 and *p* < 0.01, respectively). Scale bars, 200 µm.

The SeC-dependent increase in cell numbers in healthy and DMD myoblasts was maintained at day 6 in DM, and was particularly evident in Del51 myoblasts, as observed by phase contrast microscopy (Figure S1A) and evaluated by counting the total nuclei after May–Grünwald/Giemsa staining (~52% increase in total nuclei in the presence of SeC compared with control) (Figure 4A,B). At this time-point, co-culturing healthy myoblasts with SeC did not affect apparently the final fusion extent into myotubes, whereas DMD myoblasts seemed have had benefit from the presence of SeC in terms of myotube formation. Indeed, co-culture with SeC did not affect fusion indexes in healthy myoblasts, and increased fusion indexes in DMD myoblasts, with Del51 showing ~54% increase (Figure 4C). In accordance, at day 6 in DM, DMD, but not healthy myotubes, showed significantly higher nuclei/myotube ratios when co-cultured with SeC (~69% increase in the case of Del51) (Figure 4D).

Analysis after immunofluorescence staining for MyHC-II showed similar average myotube diameters in the absence or presence of SeC in both healthy and DMD myotubes (Figure 4E,F). Together with the increased nuclei/myotube ratios (Figure 4D), this suggests that factors secreted by SeC sustain the longitudinal growth (elongation) in DMD myotubes.

**Figure 5:**
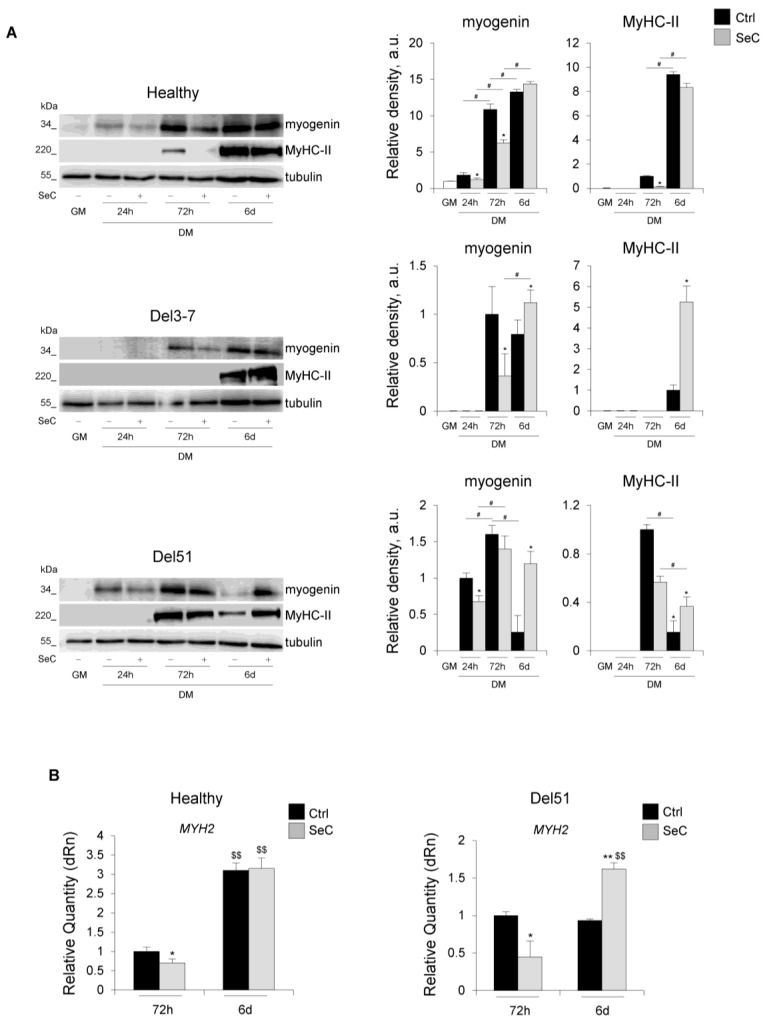
SeC-treated DMD myoblasts show delayed early and improved terminal differentiation. (**A**,**B**) Human myoblasts derived from healthy donor and DMD (Del3-7 and Del51) patients were co-cultured in GM for 24 h or in DM with (SeC) or without (Ctrl) freshly-isolated SeC (2.0 × 10^5^ SeC/mL) using 0.4 μm BD transwells for 24 h, 72 h or 6 days. (**A**) Cells were lysed and the expression of myogenin and myosin heavy chain (MyHC)-II were analyzed by WB. Representative images of three independent experiments are reported. The average relative densities (±SD) of the bands were determined with respect to tubulin bands, used as house-keeping protein. (**B**) Real-time PCR analysis for the expression of *MYH2* (MyHC) in healthy and Del51 subjects at 72 h and 6 days. Reported are the means (±SD) with respect to control (Ctrl) at 72 h and normalized for *GAPDH* used as house-keeping gene. * and **, significantly different from Ctrl (*p* < 0.05 and *p* < 0.01, respectively); #, significantly different (*p* < 0.05); $$, significantly different from the same treatment at 72 h (*p* < 0.01).

In order to have information about the effects of SeC on the muscle differentiation process at molecular level, we investigated the expression of the muscle-specific markers, myogenin and MyHC-II. In the presence of SeC, the myogenin amounts were reduced in both healthy and DMD (Del51 and Del3-7) myoblasts at 24 h and/or 72 h in DM, and unchanged or increased in healthy and DMD myoblasts, respectively, at 6 days in DM in comparison with the corresponding untreated cells (Figure 5A). A similar expression pattern emerged for MyHC-II, whose expression was delayed until 72 h in the presence of SeC, and whose amounts were found unchanged or increased at 6 days in healthy and DMD myotubes, respectively (Figure 5A), in accordance with the respective fusion indexes (Figure 4C). This was corroborated by real-time PCR analysis showing lower expression levels of the MyHC-II gene (*MYH2*) at 72 h in DM in healthy and Del51 myoblasts co-cultured with SeC (Figure 5B). However, at 6 days in DM *MYH2* expression was found unchanged in healthy and dramatically increased in Del51 myoblasts (Figure 5B), in accordance with the detected protein amounts.

Thus, in DMD myoblasts co-cultured with SeC an early increase in cell numbers occurs that is followed by enhanced differentiation.

### 3.3. SeC Restrain the Fibrogenic Potential of Fibroblasts and Inhibit Myoblast–Myofibroblast Transdifferentiation

**Figure 6:**
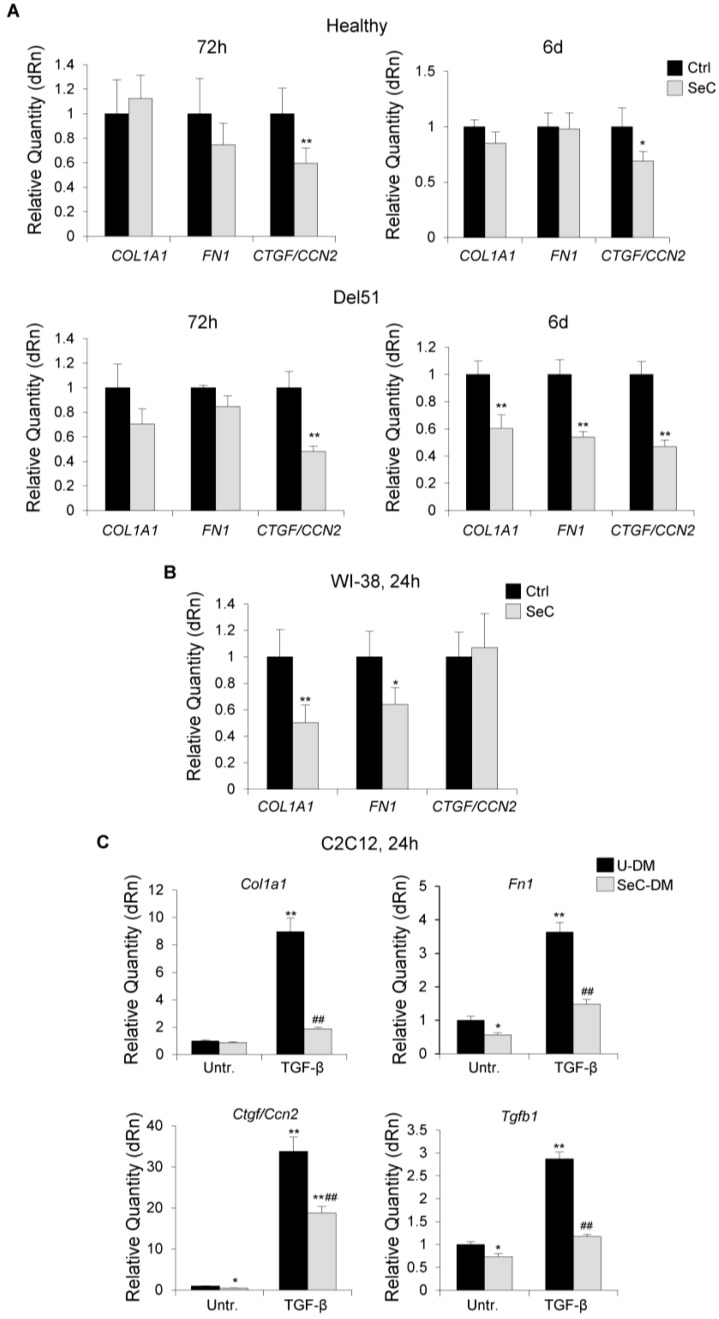
SeC restrain the fibrogenic potential of fibroblasts and inhibit fibrogenic transdifferentiation of myoblasts. (**A**,**B**) Human myoblasts derived from healthy donor and Del51 patient (**A**) or human WI-38 fibroblasts (**B**) were co-cultured in DM with (SeC) or without (Ctrl) freshly-isolated SeC (2.0 × 10^5^ SeC/mL) using 0.4 μm BD transwells at the indicated times. Cells were lysed and analyzed for the expression of the fibrogenic markers, *COL1A1*, *FN1,* and *CTGF*/*CCN2* by real time-PCR. (**C**) C2C12 myoblasts cultured in SeC-conditioned (SeC-DM) or unconditioned (U-DM) medium were induced to transdifferentiate into myofibroblasts by treatment with TGF-β (5 ng/mL) for 24 h. Cells were lysed and analyzed for the expression of *Col1a1*, *Fn1*, *Ctgf*/*Ccn2*, and *Tgfb1* by real time-PCR. Reported are the means (±SD) of three independent experiments with respect to control (Ctrl) normalized for *GAPDH* (**A**,**B**) or *Gapdh* (**C**) used as house-keeping genes. * and **, significantly different from Ctrl or untreated U-DM (Untr.) (*p* < 0.05 and *p* < 0.01, respectively); ##, significantly different from TGF-β-treated U-DM (*p* < 0.01).

DMD muscles are characterized by progressive deposition of fibrotic tissue, which has been linked in part to the transdifferentiation of dystrophic myoblasts into myofibroblasts. Indeed, DMD myoblasts were found to express a more fibrogenic phenotype compared with healthy ones [21]. We found that Del51 myoblasts co-cultured with SeC for 6 days in DM expressed lower levels of the fibrotic markers, *COL1A1* (collagen 1A1), *FN1* (fibronectin) and *CTGF/CCN2* (connective tissue growth factor/cellular communication network factor 2) (Figure 6A), which play a major role in fibrogenesis, suggesting that SeC-secreted factors might inhibit the appearance of the fibrogenic phenotype in DMD muscles. Among the markers tested, *CTGF*/*CCN2* was the only one whose expression was reduced by SeC in Del51 myoblasts already at 72 h and in healthy myoblasts irrespective of the time-point considered (Figure 6A).

Since myoblast primary cultures are likely to contain a fibroblastic component at different extent, which could mask or concur to the observed effects of SeC on human myoblasts, we performed experiments with a purified fibroblast cell line. Human WI-38 fibroblasts showed significantly reduced expression of the profibrotic genes, *COL1A1* and *FN1* when co-cultured with SeC in comparison with control fibroblasts (Figure 6B), indicating a direct effect of SeC on the fibroblastic component. However, *CTGF*/*CCN2*, which was the most downregulated fibrogenic marker in myoblast primary cultures (Figure 6A), resulted not significantly affected by SeC in fibroblasts (Figure 6B), suggesting a direct effect of SeC on myoblasts.

To further investigate the role of SeC in myoblast transdifferentiation, we treated the stabilized myoblast cell line, C2C12, with TGF-β, which is known to induce transdifferentiation of myoblasts into myofibroblasts [22] and has a major role in inducing fibrosis in DMD muscles [23], in the absence or presence of SeC-DM. We found that SeC-derived factors dramatically hampered the expression of TGF-β-induced *Col1a1*, *Fn1*, *Ctgf*/*Ccn2*, and *Tgfb1* itself (Figure 6C).

Thus, SeC appear able to both protect myoblasts against myofibroblast transdifferentiation and restrain the fibrogenic potential of preexisting fibroblasts.

### 3.4. SeC Induce Utrophin Expression in DMD Myotubes Regardless of the Mutation

**Figure 7:**
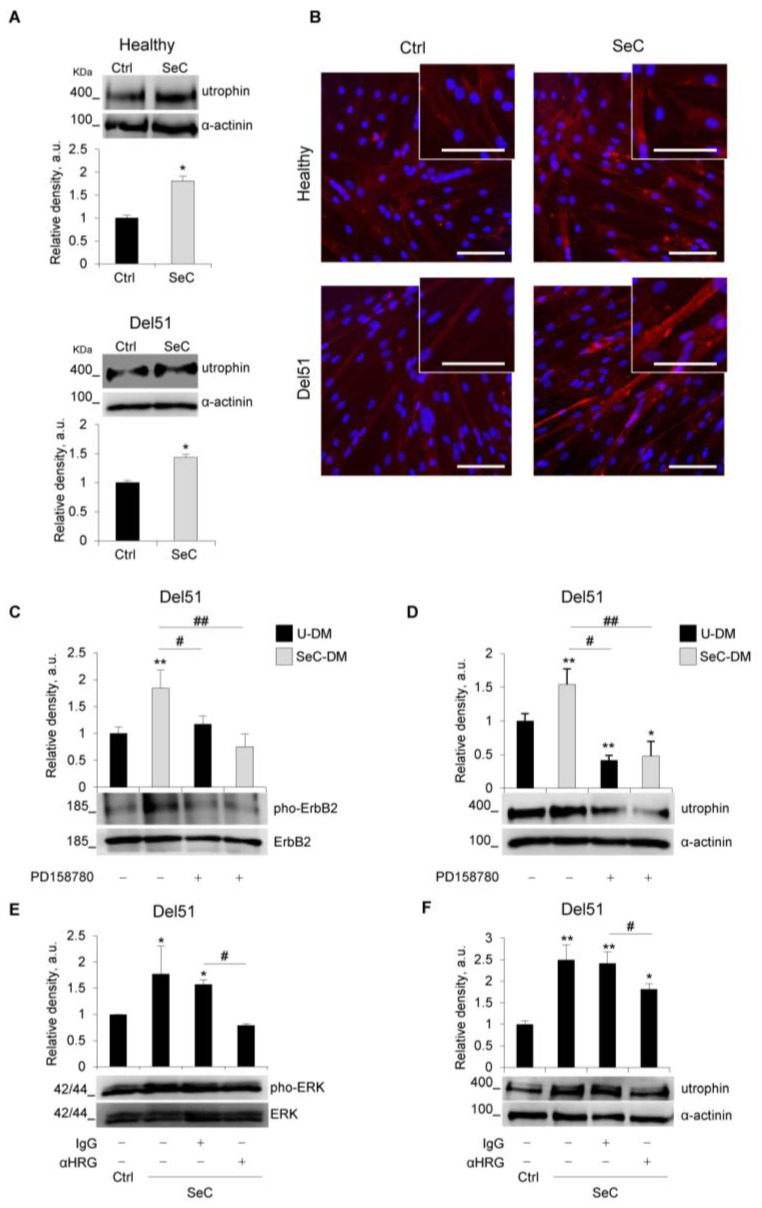
SeC up-regulate utrophin expression in human healthy and DMD myotubes. (**A**–**F**) Human myotubes obtained by culturing myoblasts derived from healthy donor or Del51 patient in differentiation medium for 4 days were co-cultured in the absence (Ctrl) or presence of SeC (2.0 × 10^5^ SeC/mL) using 0.4 μm transwells (**A**,**B**,**E**,**F**), or cultured in SeC-conditioned (SeC-DM) or unconditioned (U-DM) differentiation medium (**C**,**D**). (**A**) After 48 h, myotubes were lysed and analyzed for utrophin expression by WB. The average relative densities of utrophin bands with respect to α-actinin bands are reported. (**B**) Immunofluorescence analysis for utrophin (red) expression was performed at 48 h, and DAPI (blue) was used to counterstain nuclei. (**C**,**D**) Myotubes were cultured with SeC-DM or U-DM for 24 h in the absence or presence of the ErbB receptor tyrosine kinase inhibitor, PD158780 (100 nM) or the same amounts of vehicle (DMSO). Myotubes were lysed and utrophin (**C**) and phosphorylated ErbB2 (pho-ErbB2) (**D**) expression were analyzed by WB. The average relative densities of utrophin and pho-ErbB2 bands with respect to α-actinin or total ErbB2 bands, respectively, are reported. (**E**,**F**) Myotubes co-cultured with or without SeC were added with either an anti-heregulin β1 blocking antibody (αHRG, 2 μg/mL) or the same amounts of non-immune IgG to the upper chamber of the transwells for 24 h (**E**) or 48 h (**F**). Myotubes were lysed and phosphorylated ERK1/2 (pho-ERK) (**E**) and utrophin (**F**) expression were analyzed by WB. The average relative densities of pho-ERK and utrophin bands with respect to total ERK or α-actinin bands, respectively, are reported. * and **, significantly different from Ctrl or untreated U-DM (*p* < 0.05 and *p* < 0.01, respectively). # and ##, significantly different (*p* < 0.05 and *p* < 0.01, respectively). Reported are representative images (**A**–**F**). Results are means (±SD) of three independent experiments. Scale bars (**B**), 50 µm.

Previous work demonstrated that SeC induce the expression of utrophin in murine dystrophic muscles, contributing to the SeC-dependent rescue of muscle morphology and performance [12]. To investigate a similar effect in humans, preformed myotubes obtained from healthy subject or DMD patients (Del51, Del3-7, and G > T transition in the exon 23) were co-cultured with freshly purified SeC. After 48h, we found an increased expression of utrophin in both healthy and DMD myotubes, ranging from 1.4- to 2.3-fold increase compared with untreated controls (Figure 7A and Figure S2A). In accordance, immunofluorescence staining for utrophin revealed a more intense fluorescence signal in myotubes cultured in the presence of SeC, and showed that utrophin was mainly located at the sarcolemma (Figure 7B and Figure S2B). Interestingly, the expression of the member of the utrophin-associated protein complex (UAPC), α-dystroglycan was found also increased at the periphery of healthy and DMD myofibers in the presence of SeC (Figure S3).

Del51 myotubes were further investigated for the dependence of utrophin induction by SeC-secreted heregulin β1. We found that SeC-DM induced tyrosine phosphorylation of ErbB2 receptor (Figure 7C), whose heterodimerization with ErbB3 is induced by heregulin β1 leading to receptor tyrosine phosphorylation and activation of downstream signal transduction [24], and induction of utrophin expression (Figure 7D). Both ErbB2 phosphorylation and utrophin induction were abrogated by the use of PD158780, a potent cell-permeable ErbB receptor tyrosine kinase inhibitor (Figure 7C,D). Moreover, the induction of utrophin was accompanied by activation (phosphorylation) of ERK1/2 MAPKs (Figure 7E), and the use of an anti-heregulin β1 antibody hampered the ability of SeC to induce utrophin expression (Figure 7F), and completely abolished the SeC-induced phosphorylation of ERK1/2 MAPKs (Figure 7E). Altogether, these data suggest that similar mechanisms to those reported in mice [12] underpin the SeC-dependent induction of utrophin in human myotubes.

## 4. Discussion

Sertoli cells (SeC) continue to unravel their peculiar effects on a growing number of physiological and pathological conditions [16,25]. In addition to their role in creating a physical barrier in the testes necessary for spermatogenesis, SeC secrete a plethora of factors with trophic and anti-inflammatory effects that have prompted researchers to evaluate the beneficial use of these cells in many pre-clinical models of diseases. Depending on the experimental model investigated, SeC have shown the ability to protect allogeneic or xenogeneic grafts from immune rejection, or to reduce local inflammation [14,16,26]. Animal models of type 1 or type 2 diabetes, Laron syndrome (dwarfism), Huntington’s disease, and others, have been reported to take advantage of the injection of naked or microencapsulated (MC-) SeC [14,25,27,28,29,30].

Duchenne muscular dystrophy (DMD) is a lethal genetic disease characterized by progressive muscle degeneration due to lack of functional dystrophin. DMD muscles are characterized by continuous necrosis of myofibers leading to a persistent inflammatory state, and loss of muscle mass and strength due to the accumulation of fibrotic and adipose tissues over time [3,31]. The *DMD* gene is the largest human gene (2.4 Mb), including 79 exons and representing 0.08% of the human genome. The very large size of the *DMD* gene and the limited homing to muscles demonstrated by ex vivo corrected cells represent the major obstacles encountered by gene- and cell-based therapeutic approaches, respectively [8,32,33,34]. Another problem is represented by the need of immunosuppression in most proposed approaches to DMD, including therapies with antisense oligonucleotides (AONs), which have also the limitation of being useful only for specific *DMD* mutations [3,34]. Despite a huge effort in finding a cure, the therapeutic approaches to DMD proposed so far have resulted unsatisfactory in terms of functional recovery, leading to the idea that a combinatorial approach is needed.

MC-SeC injected into the peritoneal cavity of dystrophic (*mdx*) mice proved to be beneficial in recovering muscle homeostasis thanks to the release of a cocktail of factors that counteract muscle inflammation and fibrosis, and the induction of the expression of the dystrophin paralogue, utrophin at the sarcolemma of myofibers [11,12]. However, data about the effects of SeC on satellite cells (i.e., the adult stem cells of skeletal muscle residing between the basal lamina and the plasmalemma [35]) and their progeny, the myoblasts, were not available. Here, we demonstrate that SeC have direct effects on myoblasts, sustaining their proliferation (and inhibiting ROS production and the apoptotic events in the case of C2C12 myoblasts) in the early phase of the differentiation process. Concomitantly, SeC do not affect (in the case of healthy human myoblasts) or even improve (in the case of C2C12 and DMD myoblasts) the myogenic terminal differentiation. The improved differentiation capability observed in the presence of SeC might be, at least in part, the result of increased myoblast survival favoring the achievement of the critical cell mass necessary to give rise to cell fusion and differentiation.

The effect in increasing cell numbers is of particular importance when looking at DMD patients, in which a progressive emptying of the satellite cell pool occurs as an outcome of the pathology due to the continuous degeneration/regeneration cycles, leading to defective muscle repair and favoring fibrotic and adipose tissue accumulation [6,36]. Incidentally, our data suggest that SeC-based approaches might be useful in improving the early phase of muscle regeneration, during which myoblasts have to adequately proliferate to replace the damaged muscle mass [37].

Noteworthy, the most relevant effect of SeC in improving myogenic differentiation was seen in DMD myoblasts. Contrary to healthy myoblasts, an inability to upregulate *MYH2* transcript levels at day 6 in DM emerged in Del51 myoblasts in basal conditions; however, this was rescued by the presence of SeC, which elicited a three-fold upregulation of *MYH2* expression together with a dramatic increase in MyHC-II protein amounts (Figure 5A,B). This is another interesting effect in consideration of an application of SeC-based treatments to DMD patients.

Several factors known to be secreted by SeC [8,16] might participate to the effects we observed on myoblast proliferation and differentiation. They include, but are not limited to, bFGF (basic fibroblast growth factor) [38], BMP (bone morphogenetic protein) [39], IGFs (insulin-like growth factors) [40], EGF (epidermal-derived growth factor) [41,42], prosaposin [43], and TGF-β [44]. The precise combination of these factors, together with other (also still unknown) factors secreted by SeC, is the responsible of the effects exerted by SeC on healthy and DMD myoblasts.

Fibrosis is a typical hallmark of DMD muscles, and it is mainly linked to excess expression and activity of TGF-β [23]. Transdifferentiation of myogenic cells into myofibroblasts appears to contribute to the development of fibrosis in injured muscles [45]. We found that during the myogenic differentiation process SeC restrain the expression of fibrogenic markers, especially *CTGF*/*CCN2*, a cytokine synthesized by myoblasts and myotubes in response to TGF-β and responsible for TGF-β fibrotic activity through increased expression of *COL1A1* [46]. This represents a paradigm of the activity of SeC, which despite secreting TGF-β [16,26] exert an overall antifibrotic effect as a resultant of the cocktail of factors secreted. This was particularly stressed by the results obtained with C2C12 myoblasts showing that SeC are even able to counteract the activity of exogenously administered fibrogenic doses of TGF-β. The SeC-dependent reduction of *CTGF*/*CCN2* levels might be also functional to improve the myogenic potential since *CTGF*/*CCN2* has been reported to partially inhibit skeletal muscle differentiation and dedifferentiate committed myoblasts [47]. Moreover, our data show that SeC are able to restrain the fibrogenic potential of fibroblasts, thus revealing another property of SeC useful in counteracting fibrosis in DMD muscles.

In our experimentation, we found that the use of SeC-conditioned medium (SeC-DM) gave very similar results to freshly-isolated SeC in terms of increased cell numbers and improved myogenic differentiation, in both C2C12 and human myoblasts. However, in comparison with SeC-DM, freshly-isolated SeC exerted a more pronounced promitogenic effect and slightly delayed the appearance of differentiation markers (at 24 h in DM), although culminating in improved muscle differentiation at 72 h (data not shown). Thus, treatments centered on acellular SeC derivatives can be envisaged to overcome ethical issues linked to the use of living cells. Since extracellular vesicles have been reported as having a crucial role in intercellular communication through incorporation of their cargo into target cells, regulating a multitude of physiological and pathological processes [48], future studies should investigate the therapeutic potential of SeC-derived extracellular vesicles.

Due to its large size, the *DMD* gene is particularly susceptible to spontaneous mutations. About 7000 different *DMD* mutations have been reported, 90% of which translate into disruption of the open reading frame with appearance of the Duchenne phenotype [49]. Re-expression of utrophin is one of the investigated universal (i.e., suitable regardless of the mutation) approaches to treat DMD muscles since utrophin can serve as a surrogate for dystrophin due to functional redundancy between these two proteins. Indeed, utrophin is able to recruit at the plasmalemma a UAPC that shares members with the dystrophin-associated protein complex (DAPC) recruited by dystrophin in normal conditions, and exerts similar protective functions [50]. Our results show that SeC are able to induce utrophin expression in human DMD myotubes with different mutations, with a mechanism driven by porcine heregulin β1, and involving ErbB2 and ERK1/2 MAPK phosphorylation. As reported for *mdx* mice [12], SeC-induced utrophin is sufficient to recruit members of the UAPC at the periphery of human DMD myotubes, pointing to a functional replacement of the missing dystrophin.

Through activation of ErbB2, SeC-derived heregulin β1 might concur also to the observed increased numbers of myoblasts since it has been reported that muscle-specific ErbB2 knockout mice show defects in muscle repair due to decreased myoblast viability during the differentiation process [51].

Together with the reported secretion of trophic and anti-inflammatory factors by SeC in dystrophic mice [11,12] and no need of immunosuppression typical of SeC-based treatments [12,16,26,27,28,29,30], the current data support a potential use of SeC as universal (i.e., mutation-independent) approach to DMD. Indeed, we show here that SeC (i) stimulate the proliferation and subsequent terminal differentiation of dystrophic myoblasts; (ii) restrain the fibrogenic potential of fibroblasts, and reduce myoblast-myofibroblast transdifferentiation; and, (iii) induce utrophin expression in myotubes/myofibers irrespective of the *DMD* mutation.

This is an in vitro study on a murine cell line and a limited number of human-derived healthy and DMD myoblasts that expands an in vivo study in an animal model of DMD [11,12]. The SeC secretory activity results in the production of a cocktail of factors whose formulation is difficult to dissect, the overall effect being not necessarily the sum of the biological effects of single factors [8,25,26]. The identification of a minimal cocktail of factors able to mimic the reported effects of SeC on myoblasts/myotubes should be the aim of further studies.

## Data Availability

No new data were created or analyzed in this study. Data sharing is not applicable to this article.

## Supplementary Materials

The following are available online at https://www.mdpi.com/article/10.3390/biom11101504/s1, Figure S1: SeC promote early cell proliferation and late terminal differentiation in human healthy and DMD myoblasts, Figure S2: SeC up-regulate utrophin expression in DMD myotubes, Figure S3: α-Dystroglycan is recruited at the periphery of healthy and DMD myotubes co-cultured with SeC.

## Appendix A

**Table A1:** Key resources table.

Reagent or Resource	Source	Identifier
**Antibodies**		
Donkey anti-Mouse IgG (H + L), Alexa Fluor 488 conjugated	Thermo Fisher Scientific	Cat#A-21202
Donkey anti-Mouse IgG (H + L), Alexa Fluor 594 conjugated	Thermo Fisher Scientific	Cat#A-21203
Goat anti-mouse IgG/IgM-HRP conjugated	Merck	Cat#AP130P
Goat anti-rabbit IgG-HRP conjugated	Sigma-Aldrich	Cat#A9169
IgG from rabbit serum	Sigma-Aldrich	Cat#I5006
Mouse monoclonal anti- α-Dystroglycan (IIH6)	Santa Cruz Biotechnology	Cat# sc-53987
Mouse monoclonal anti-Myogenin (F5D)	Santa Cruz Biotechnology	Cat#sc-12735
Mouse monoclonal anti-Utrophin (8A4)	Santa Cruz Biotechnology	Cat#sc-33700
Mouse monoclonal anti-α-Actinin (H-2)	Santa Cruz Biotechnology	Cat#sc-17829
Mouse monoclonal anti-α-Tubulin (DM1A)	Santa Cruz Biotechnology	Cat#sc-32293
Rabbit polyclonal anti-HER2/ErbB2	Cell Signaling Tech	Cat#2242
Rabbit polyclonal anti-Heregulin-beta1	ProSci	Cat#38-254
Rabbit polyclonal anti-MAP Kinase (ERK-1, ERK-2)	Cell Signaling Tech	Cat#M5670
Rabbit polyclonal anti-phospho-ErbB2 (HER-2) (Tyr877)	Invitrogen	Cat#PA5-77970
Rabbit polyclonal anti-phospho-p44/42 MAPK (Erk1/2) (Thr202/Tyr204)	Cell Signaling Tech	Cat#9101S
**Chemicals, Peptides, and Recombinant Proteins**		
Blotto, non-fat dried milk	Santa Cruz Biotechnology	Cat#sc-2325
Bovine Serum Albumin (BSA)	Sigma-Aldrich	Cat#A7030
DAPI dihydrochloride	Sigma-Aldrich	Cat#D9542
DCFH-DA (2′,7′-Dichlorofluorescin diacetate)	Sigma-Aldrich	Cat#D6883
DMSO (Dimethyl sulfoxide)	Sigma-Aldrich	Cat#67-68-5
Gelatin solution	Sigma-Aldrich	Cat#G1393
Giemsa stain, modified	Sigma-Aldrich	Cat#GS500
May–Grünwald stain	Sigma-Aldrich	Cat#MG500
PD 158780 tyrosine kinase inhibitor	Santa Cruz Biotechnology	Cat#CAS 171179-06-9
Propidium iodide	Sigma-Aldrich	Cat#81845
Recombinant human TGF-β	R&D System	Cat#240-B
TRIsure ™	Bioline	Cat#BIO-38033
Western Bright Quantum HRP substrate	Advansta	Cat#K-12042-D20
**Experimental Models: Cell Lines**		
C2C12 mouse myoblasts	ATCC, American Type Culture Collection	Cat#CRL-1772
WI-38 human fibroblasts	ATCC, American Type Culture Collection	Cat#CCL-75
**Culture Medium**		
Apo-Transferrin human	Sigma-Aldrich	Cat#T1147
Collagenase P	Sigma Aldrich	Cat#11213865001
DNase I	Sigma Aldrich	Cat #SLCB2343
Fetal bovine serum (FBS)	Gibco	Cat#10270-106
Gentamicin 10 mg/mL sulphate	Euroclone	Cat#ECM0011B
HAM’S F12	Euroclone	Cat#AL025A
High-glucose Dulbecco’s Modified Eagle’s Medium (HG-DMEM)	Gibco	Cat#41966-029
Horse Serum (HS)	Gibco	Cat#16050-122
Insulin solution from bovine pancreas	Sigma-Aldrich	Cat#I0516-5ML
Insulin-Transferrin-Selenium (ITS) + Premix	Corning	Cat#354352
L-Glutamine 100× (200 mM)	Euroclone	Cat#ECB3000D
Penicillin/Streptomicin 100×	Euroclone	Cat#CB3001D
Retinoic acid	Sigma-Aldrich	Cat#R-2625100
RPMI Medium 1640	Gibco	Cat#21875-034
Skeletal Muscle Cell Growth Medium (ready-to-use kit)	Promo Cell	Cat#39365
Trypsin (2.5%)	Gibco	Cat#15090-046
**Oligonucleotides**		
Primers (see Appendix B)	Invitrogen	N/A
**Software and Algorithms**		
IBM^®^ SPSS^®^ Statistics v18 software	SPSS (Chicago, IL, USA)	
Image Studio Digits v3.1.4	LI-COR	
ImageJ v1.8.0_172	Wayne Rasband (NIH, USA)	
LAS (Leica Application Suite) software v4.12.0	LEICA	
MxPro-Mx3000P v4.10	Agilent Technologies Stratagene	
SPOT Imaging v3.5.4	Diagnostic Instruments	
**Other**		
Annexin V-FITC apoptosis detection kit	BioVision Inc.	K101-100
5× HOT FIREPol EvaGreen qPCR Mix Plus (ROX)	Solis BioDyne	08-24-0000
PrimeScript ™ RT reagent Kit with gDNA Eraser	Takara Bio Europe	RR047B

## Appendix B

**Table A2:** List of primary and secondary antibodies used in Western blotting.

Primary Antibody	Molecular Weight (kDa)	Dilution	Secondary Antibody	Dilution
Mouse monoclonal anti-MyHC-II (MF20)	220	1:10,000	Goat anti-mouse IgG/IgM-HRP conjugated	1:10,000
Mouse monoclonal anti-myogenin (F5D)	34	1:1000	Goat anti-mouse IgG/IgM-HRP conjugated	1:1000
Mouse monoclonal anti-Neu (ErbB2) (3B5)	185	1:200	Goat anti-mouse IgG/IgM-HRP conjugated	1:1000
Mouse monoclonal anti-utrophin (8A4)	400	1:1000	Goat anti-mouse IgG/IgM-HRP conjugated	1:1000
Mouse monoclonal anti-α-actinin (H-2)	100	1:5000	Goat anti-mouse IgG/IgM-HRP conjugated	1:5000
Mouse monoclonal anti-α-tubulin (DM1A)	55	1:5000	Goat anti-mouse IgG/IgM-HRP conjugated	1:5000
Rabbit polyclonal anti-MAP kinase (ERK1, ERK2)	42–44	1:10,000	Goat anti-rabbit IgG-HRP conjugated	1:10,000
Rabbit polyclonal anti-phospho-ErbB2 (HER-2) (Tyr877)	185	1:500	Goat anti-rabbit IgG-HRP conjugated	1:2000
Rabbit polyclonal anti-phospho-p44/42 MAPK (ERK1/2) (Thr202/Tyr204)	42–44	1:1000	Goat anti-rabbit IgG-HRP conjugated	1:2000

## Appendix C

**Table A3:** List of primers used in real-time PCR.

Gene	Forward Primer 5′-3′	Reverse Primer 5′-3′
*COL1A1*	TCTGCGACAACGGCAAGGTG	GACGCCGGTGGTTTCTTGGT
*CTGF*/*CCN2*	CTTGCGAAGCTGACCTGGAAGA	CCGTCGGTACATACTCCACAGA
*FN1*	ACAACACCGAGGTGACTGAGAC	GGACACAACGATGCTTCCTGAG
*GAPDH*	AAGAAGGTGGTGAAGCAGG	GTCAAAGGTGGAGGAGTGG
*MYH2*	CAGCTACTGCACACCCAGAA	CTTCACGGTCTGCTCCATGT
*Col1a1*	TCATCGTGGCTTCTCTGGTC	GACCGTTGAGTCCGTCTTTG
*Ctgf*/*Ccn2*	GCCTACCGACTGGAAGACAC	GTAACTCGGGTGGAGATGCC
*Fn1*	GAAGACAGATGAGCTTCCCCA	GGTTGGTGATGAAGGGGGTC
*Gapdh*	GCCTTCCGTGTTCCTACCC	CAGTGGGCCCTCAGATGC
*Tgfb1*	GCCTGAGTGGCTGTCTTTTGA	CACAAGAGCAGTGAGCGCTGAA
