# Supplementary material for: Sertoli Cells Improve Myogenic Differentiation, Reduce Fibrogenic Markers, and Induce Utrophin Expression in Human DMD Myoblasts
Source: Biomolecules. 2021 Oct 12;11(10):1504. doi: 10.3390/biom11101504 (PMC8533898; doi:10.3390/biom11101504)
Supplement: Supplementary file 1 [file biomolecules-11-01504-s001.zip › Biomolecules Supplementary Figures.pdf]

Supplementary figures

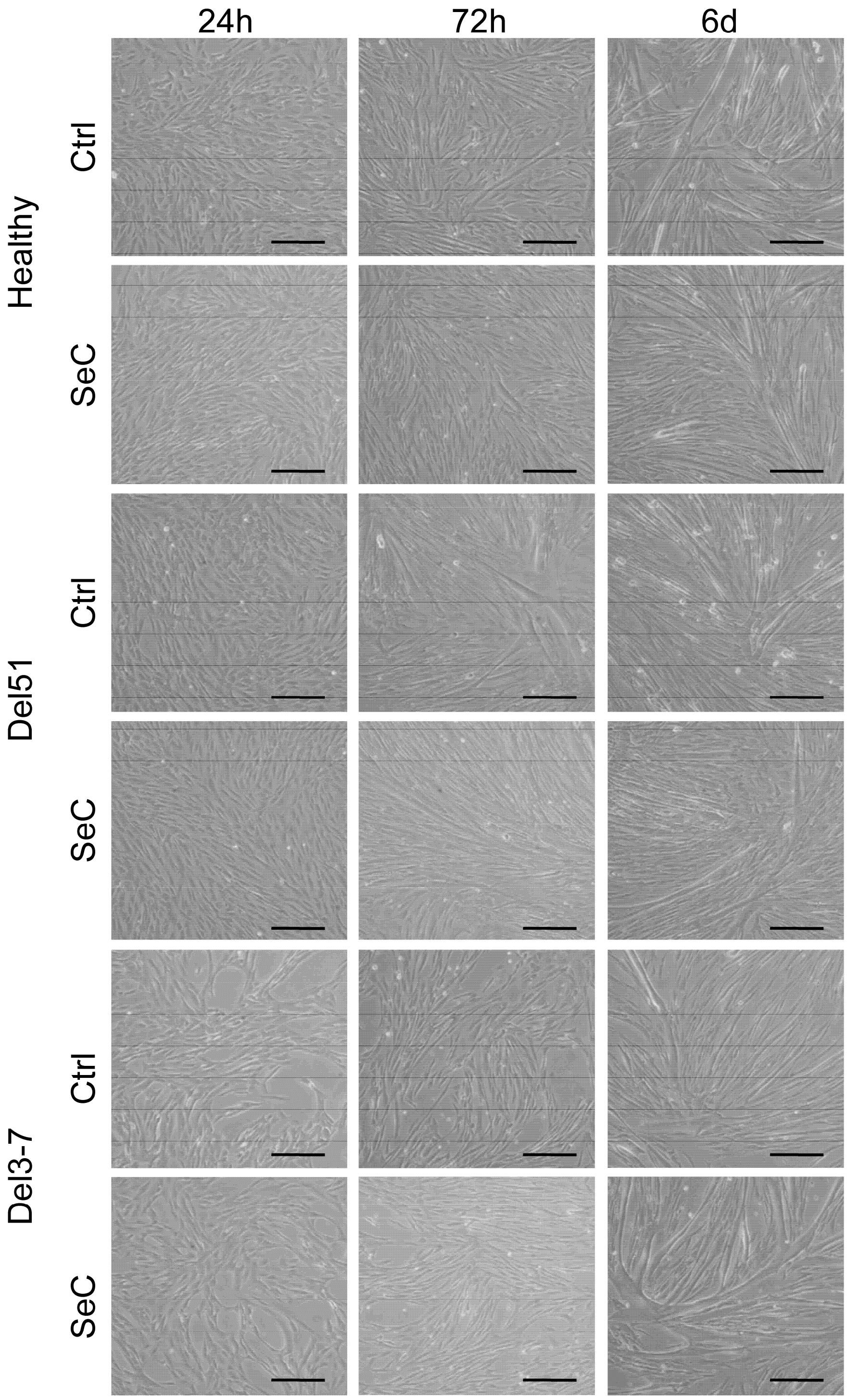

Figure S1. SeC promote early cell proliferation and late terminal differentiation in human healthy and DMD myoblasts. Reported are representative phase contrast microscopy images of human myoblasts derived from healthy donor and DMD (Del3-7 and Del51) patients co-cultured in DM with or without freshly-isolated SeC (2.0x10^5 SeC/ml) using 0.4μm transwells for 24h, 72h and 6d. Scale bars, 200μm.

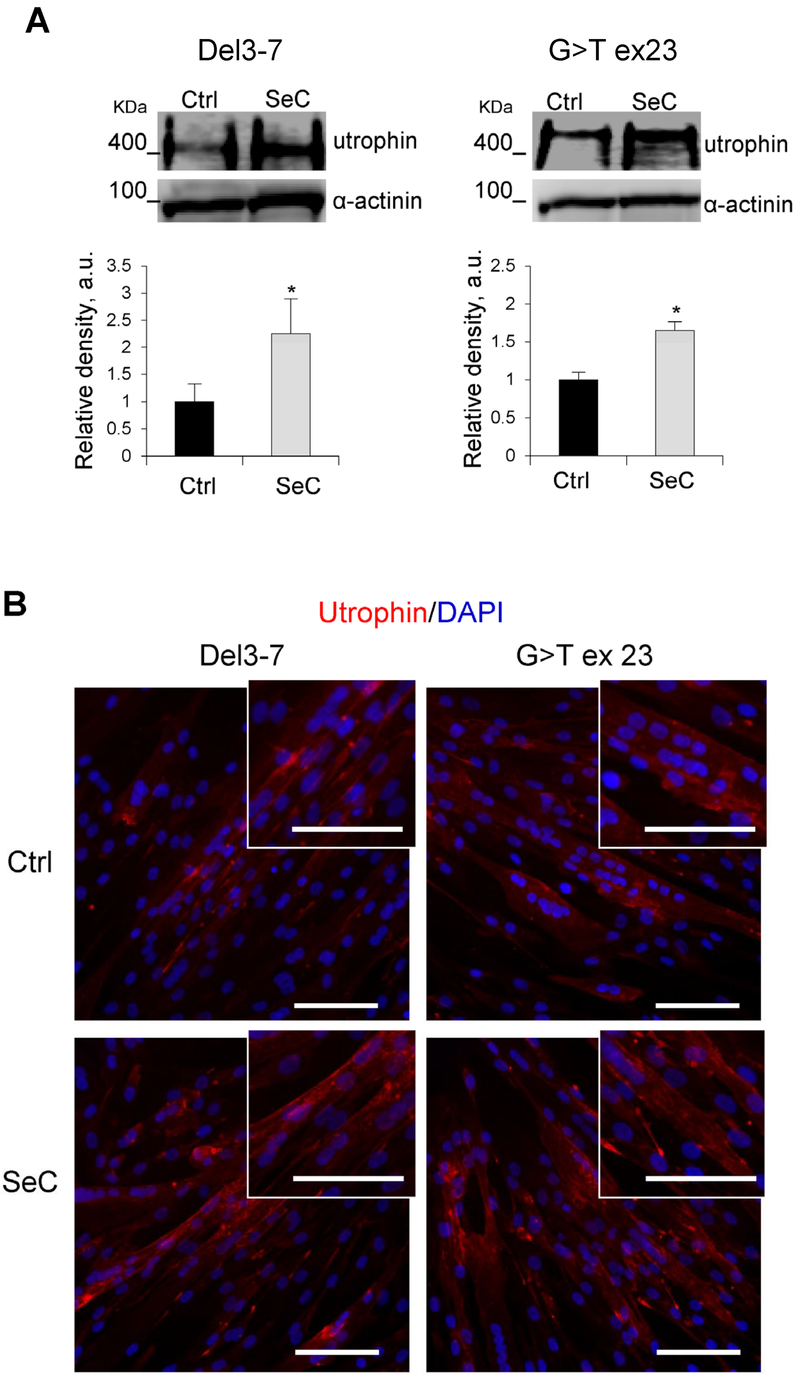

Figure S2. SeC up-regulate utrophin expression in DMD myotubes. (A-B) Myotubes obtained by culturing myoblasts derived from DMD patients [Del3-7 and G>T ex23 (G>T transition in exon 23)] in differentiation medium for 4 days were co-cultured with or without (Ctrl) SeC (2.0×10^5 SeC/ml) using 0.4μm transwells for 48h. (A) Myotubes were lysed and analyzed for utrophin expression by WB. The average relative densities of utrophin bands with respect to α-actinin bands are reported. (B) Immunofluorescence analysis for utrophin (*red*) was performed, and DAPI (*blue*) was used to counterstain nuclei. *, significantly different from Ctrl (P<0.05). Results are means (±SD) of three independent experiments. Scale bars (B), 50μm.

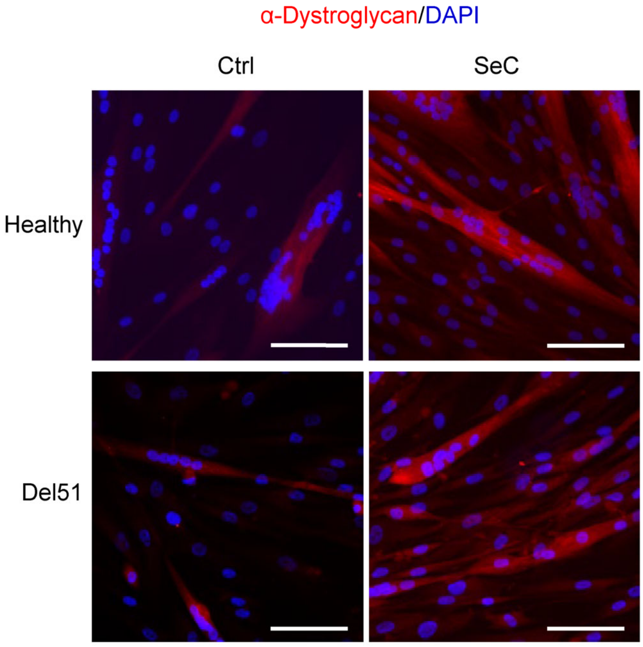

Figure S3. α-Dystroglycan is recruited at the periphery of healthy and DMD myotubes co-cultured with SeC. Myotubes obtained by culturing myoblasts derived from healthy donor or Del51 patient in differentiation medium for 4 days were co-cultured with or without (Ctrl) SeC (2.0×10^5 SeC/ml) using 0.4μm transwells for additional 48h. Immunofluorescence analysis for α-dystroglycan (*red*) was performed, and DAPI (*blue*) was used to counterstain nuclei. Shown are representative images. Scale bars, 100μm.
